# Supplementary material for: Synthetic Lethal Targeting of Mitotic Checkpoints in HPV-Negative Head and Neck Cancer
Source: Cancers (Basel). 2020 Jan 28;12(2):306. doi: 10.3390/cancers12020306 (PMC7072436; doi:10.3390/cancers12020306)
Supplement: Supplementary file 1 [file cancers-12-00306-s001.zip › cancers-672795-supplement-final/Supplementary Table S1.docx]

**Table S1.** Mutational profile of cell line models. Mutations affecting genes previously defined as most common in HNSCC are noted for the four cell models. Damaging mutations are indicated in red font; black, mutation is tolerable or significance of mutation unknown, and unclear from Annovar based analysis of prediction scores. NA, not applicable.

|  | **Gene** | **AA mut** | **CDS mut** | **Type** | **Genomic location** | **Nucleotide** | **Annot. Transcript** | **COSMIC ID** | **dbSNP ID** |
| --- | --- | --- | --- | --- | --- | --- | --- | --- | --- |
| **CAL27 Cell Line** | TP53 | p.H193L | c.A578T | Missense | 17:7674953..7674953 | NM_000546.5 | ENST00000269305.4 |  | rs786201838 |
|  | CDKN2A | p.E18* | c.G247T | Stopgain | 9:21971154..21971154 | NM_058195 | ENST00000479692.2 |  | rs121913383 |
|  | CASP8 | p.II174fs | c.699_702del | Frame shift del | 2:202137471…202137474 | NM_001080125 | ENST00000432109.2 | COSM2907087 | rs754434271 |
|  |  | p.V460V | c.C1380T | Silent | 2:202151302…202151302 | NM_033356 | ENST00000432109.2 |  |  |
|  | FAT1 |  |  |  |  |  |  |  |  |
|  | HUWE1 |  |  |  |  |  |  |  |  |
|  | HLA-A | NA | c.620-1G>C | Splice site | 6:29911898…29911898 | NM_002116 | ENST00000396634.1 |  | rs199474608 |
|  | EP300 |  |  |  |  |  |  |  |  |
|  | CREBBP | p.T2352N | c.C7055A | Missense | 16:3777879…3777879 | NM_001079846 | ENST00000262367.5 | COSM2919889 |  |
|  | HRAS |  |  |  |  |  |  |  |  |
|  | JUB |  |  |  |  |  |  |  |  |
|  | TGFBR2 |  |  |  |  |  |  |  |  |
|  | HLA-B |  |  |  |  |  |  |  |  |
|  | DYSF |  |  |  |  |  |  |  |  |
|  | KDM6A | p.E1023K | c.G3067A | Missense | X:44942724…44942724 | NM_001291418 | ENST00000377967.4 | COSM2965437 | rs1554099877 |
|  | RAC1 | p.L192L | c.G576A | Silent | 7:6442074…6442074 | NM_006908 | ENST00000348035.4 |  |  |
|  | PTEN |  |  |  |  |  |  |  |  |
|  | ZNF233 |  |  |  |  |  |  |  |  |

**Table S1.** *Cont*.

|  | **Gene** | **AA mut** | **CDS mut** | **Type** | **Genomic location** | **Nucleotide** | **Annot. Transcript** | **COSMIC ID** | **dbSNP ID** |
| --- | --- | --- | --- | --- | --- | --- | --- | --- | --- |
| **FaDu Cell Line** | TP53 | p.R116L | c.G347T | Missense | 17:7577538…7577538 | NM_001126115 | ENST00000269305.4 | COSM241994 | rs11540652 |
|  |  | NA | c.556-1G>A | Splice site | 17:7577609…7577609 | NM_001276761 | ENST00000269305.4 | COSM1725566 |  |
|  | CDKN2A | NA | c.425-1G>T | Splice site | 9:21971208…21971208 | NM_058197 | ENST00000304494.5 | COSM363012 |  |
|  | CASP8 |  |  |  |  |  |  |  |  |
|  | FAT1 | p.K3277Nfs*3 | c.9828delG | Frame shift del | 4:187532565...187532565 | NM_005245 | ENST00000441802.2 |  |  |
|  | HUWE1 |  |  |  |  |  |  |  |  |
|  | HLA-A |  |  |  |  |  |  |  |  |
|  | EP300 |  |  |  |  |  |  |  |  |
|  | CREBBP |  |  |  |  |  |  |  |  |
|  | HRAS |  |  |  |  |  |  |  |  |
|  | JUB |  |  |  |  |  |  |  |  |
|  | TGFBR2 |  |  |  |  |  |  |  |  |
|  | HLA-B |  |  |  |  |  |  |  |  |
|  | DYSF |  |  |  |  |  |  |  |  |
|  | KDM6A | p.A15_A17del | c.31_39del | In frame del | X:44732828…44732836 | NM_001291415 | ENST00000377967.4 |  |  |
|  | RAC1 |  |  |  |  |  |  |  |  |
|  | PTEN |  |  |  |  |  |  |  |  |
|  | ZNF233 |  |  |  |  |  |  |  |  |

**Table S1.** *Cont*.

|  | **Gene** | **AA mut** | **CDS mut** | **Type** | **Genomic location** | **Nucleotide** | **Annot. Transcript** | **COSMIC ID** | **dbSNP ID** |
| --- | --- | --- | --- | --- | --- | --- | --- | --- | --- |
| **SCC61 Cell line** | TP53 | p.R110L | c.G329T | Missense | 17:7579358..7579358 | NM_000546 | ENST00000620739 | COSM1646883 | rs11540654 |
|  |  | p.P72R | c.C215G | Polymorphism | 17:7579472…7579472 | NM_000546 |  | COSM3766192 | rs1042522 |
|  | CDKN2A |  |  |  |  |  |  |  |  |
|  | CASP8 |  |  |  |  |  |  |  |  |
|  | FAT1 | p.R2567H | c.G7700A | Missense | 4:187540040…187540040 | NM_005245 |  | COSM1193371 | rs116784674 |
|  | HUWE1 |  |  |  |  |  |  |  |  |
|  | HLA-A | p.D251H | c.G751C | Missense | 6:29912030…29912030 | NM_001242758 |  | COSM4006265 | rs145046067 |
|  |  | p.E87K | c.G259A | Missense | 6:29910719…29910719 | NM_001242758 |  | COSM4593854 | rs2230991 |
|  |  | p.E87D | c.G261C | Missense | 6:29910721…29910721 | NM_001242758 |  | COSM5019770 | rs199474424 |
|  | EP300 | p.I997V | c.A2989G | Missense | 22:41548008…41548008 | NM_001429 |  | COSM5009621 | rs20551 |
|  |  | p.M2015I | c.G6045A | Missense | 22:41573760…41573760 | NM_001429 |  |  | rs753072432 |
|  | CREBBP |  |  |  |  |  |  |  |  |
|  | HRAS |  |  |  |  |  |  |  |  |
|  | AJUBA | p.E310Q | c.G928C | Missense | 14:23450548…23450548 | NM_001289097 |  |  | rs117656327 |
|  | TGFBR2 | p.P129Afs*2 | c.374dupA | Frame shift ins | 3:30691871…30691871 | NM_003242 |  | COSM300123 | rs756007977 |
|  | HLA-B | p.I90M | c.C270G | Missense | 6:31324538…31324538 | NM_005514 |  | COSM5487119 | rs707909 |
|  |  | p.D54G | c.A161G | Missense | 6:31324647...31324647 | NM_005514 |  |  | rs9266183 |
|  |  | p.Q94H | c.G282C | Missense | 6:31324526...31324526 | NM_005514 |  | COSM4160815 | rs1131212 |
|  | DYSF | p.A171E | c.C512A | Missense | 2:71740897…71740897 | NM_001130455 |  |  | rs34999029 |
|  | KDM6A |  |  |  |  |  |  |  |  |
|  | RAC1 |  |  |  |  |  |  |  |  |
|  | PTEN |  |  |  |  |  |  |  |  |
|  | ZNF233 |  |  |  |  |  |  |  |  |

**Table S1.** *Cont*.

|  | **Gene** | **AA mut** | **CDS mut** | **Type** | **Genomic location** | **Nucleotide** | **Annot. Transcript** | **COSMIC ID** | **dbSNP ID** |
| --- | --- | --- | --- | --- | --- | --- | --- | --- | --- |
| **A-253 Cell Line** | TP53 | p.E48Gfs*66 | c.143delA | Frame shift del | 17:7578391…7578391 | NM_001126115 | ENST00000269305.4 |  |  |
|  | CDKN2A | p.A17Pfs*4 | c.49_61del | Frame shift del | 9:21974766…21974778 | NM_000077 | ENST00000304494.5 |  | rs587782206 |
|  | CASP8 |  |  |  |  |  |  |  |  |
|  | FAT1 |  |  |  |  |  |  |  |  |
|  | HUWE1 | p.D1193N | c.G3577A | Missense | X:53620488…53620488 | NM_031407 | ENST00000342160.3 |  | rs972796332 |
|  | HLA-A |  |  |  |  |  |  |  |  |
|  | EP300 |  |  |  |  |  |  |  |  |
|  | CREBBP | p.A2008A | c.G6024T | Silent | 16:3778910…3778910 | NM_001079846 | ENST00000262367.5 |  | rs371656213 |
|  | HRAS | p.C154C |  | Silent | 11:63235851…6323551 |  | ENST00000539221.1 |  |  |
|  | JUB |  |  |  |  |  |  |  |  |
|  | TGFBR2 | p.R537P | c.G1610C | Missense | 3:30732997…30732997 | NM_003242 | ENST00000295754.5 |  |  |
|  | HLA-B |  |  |  |  |  |  |  |  |
|  | DYSF |  |  |  |  |  |  |  |  |
|  | KDM6A |  |  |  |  |  |  |  |  |
|  | RAC1 |  |  |  |  |  |  |  |  |
|  | PTEN | p.L152P | c.T455C | Missense | 10:89692971…89692971 | NM_000314 | ENST00000371953.3 | COSM326268 | rs1554898197 |
|  | ZNF233 | p.C389R | c.T1165C | Missense | 19:44777978…44777978 | NM_001207005 | ENST00000391958.2 |  |  |
