# Supplementary material for: Synthetic Lethal Targeting of Mitotic Checkpoints in HPV-Negative Head and Neck Cancer
Source: Cancers (Basel). 2020 Jan 28;12(2):306. doi: 10.3390/cancers12020306 (PMC7072436; doi:10.3390/cancers12020306)
Supplement: Supplementary file 1 [file cancers-12-00306-s001.zip › cancers-672795-supplement-final/Supplementary Table S2.docx]

**Table S2.** Drugs used to screen HNSCC cell models. Compounds are listed, as are known targets; IC50 values for each target provided were obtained from product datasheets from vendor.

|  | **Compound** | **Target** | **Target IC50** |
| --- | --- | --- | --- |
| 1 | Docetaxel | Microtubules | 3–116 nM |
| 2 | Adavosertib | WEE1 | 5.2 nM |
| 3 | Alisertib | AURKA | 1.2 nM |
| 4 | AMG900 | pan-Aurora (A / B / C) | 5 nM / 4 nM / 1 nM |
| 5 | AZD0156 | ATM | 0.58 nM |
| 6 | AZD6738 | ATR | 74 nM |
| 7 | Barasertib | AURKB | 0.37 nM |
| 8 | BI 2536 | PLK / BRD4 | 0.83 nM / 25 nM |
| 9 | CC-115 | mTOR / DNA-PK | 21 nM / 13 nM |
| 10 | Danusertib | pan-Aurora (A / B / C) | 13 nM / 79 nM / 61 nM |
| 11 | Etoposide | Topoisomerase II | 600 nM |
| 12 | Irinotecan | Topoisomerase I | 5.17–15.8 µM |
| 13 | MK 8776 | CHK1 | 3 nM |
| 14 | Olaparib | PARP1 / 2 | 5 nM / 1 nM |
| 15 | Palbociclib | CDK4 / 6 | 11 nM / 16 nM |
| 16 | Prexasertib | CHK1 / 2 | <50 nM / <31 nM |
| 17 | Reversine | MPS1 / pan-Aurora (A / B / C) | 50–80 nM / 150 nM / 500 nM / 400 nM |
| 18 | Veliparib | PARP1 / 2 | 5.2 nM / 2.9 nM |
| 19 | VX-970 | ATR | 0.2 nM |
| 20 | ZM 447439 | AURKA / AURKB | 110 nM / 130 nM |
