# Supplementary figures and images for: Synthetic Lethal Targeting of Mitotic Checkpoints in HPV-Negative Head and Neck Cancer
Source: Cancers (Basel). 2020 Jan 28;12(2):306. doi: 10.3390/cancers12020306 (PMC7072436; doi:10.3390/cancers12020306)

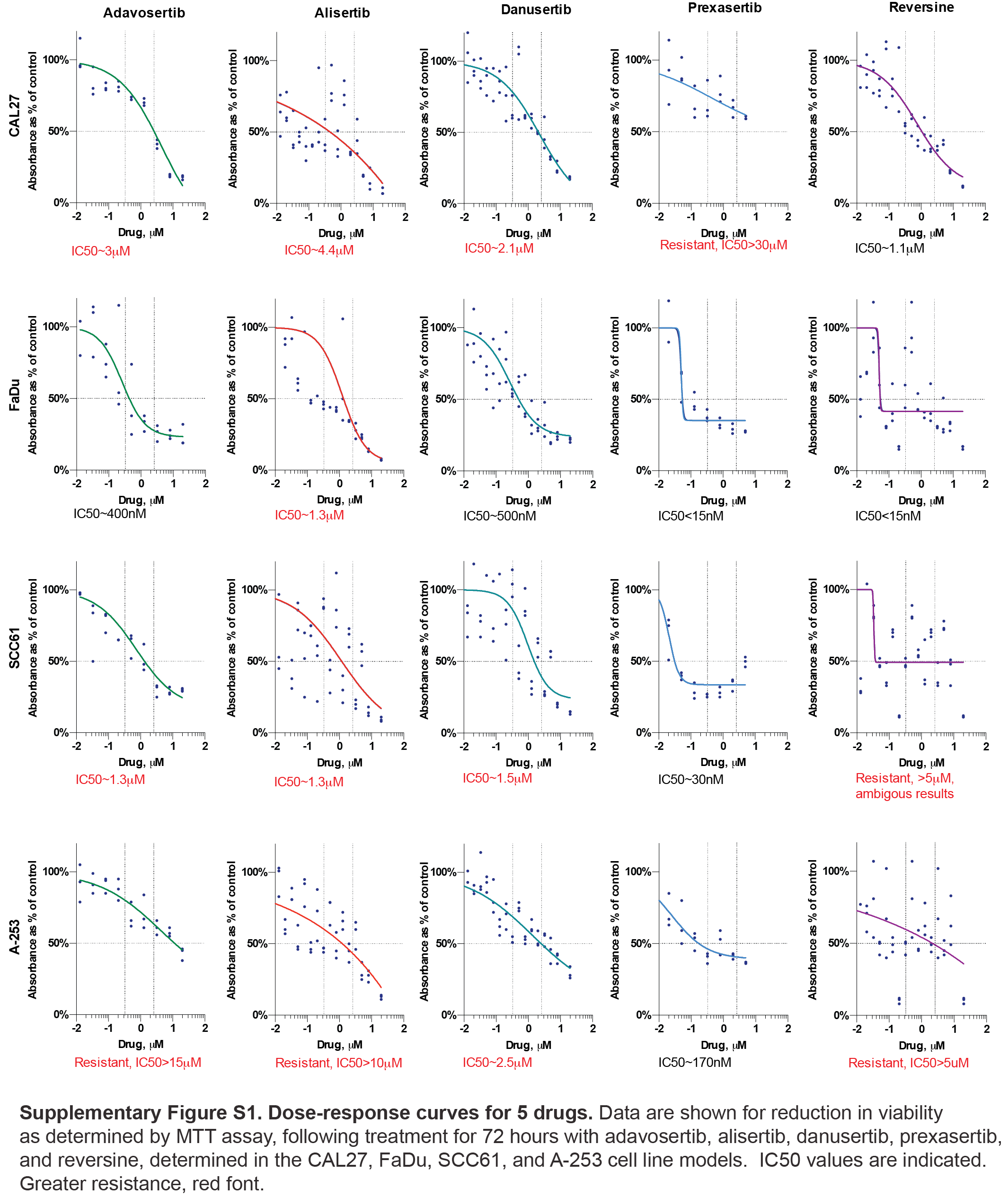

Supplement: Supplementary file 1 [file cancers-12-00306-s001.zip › cancers-672795-supplement-final/Supplementary Figure S1.png]

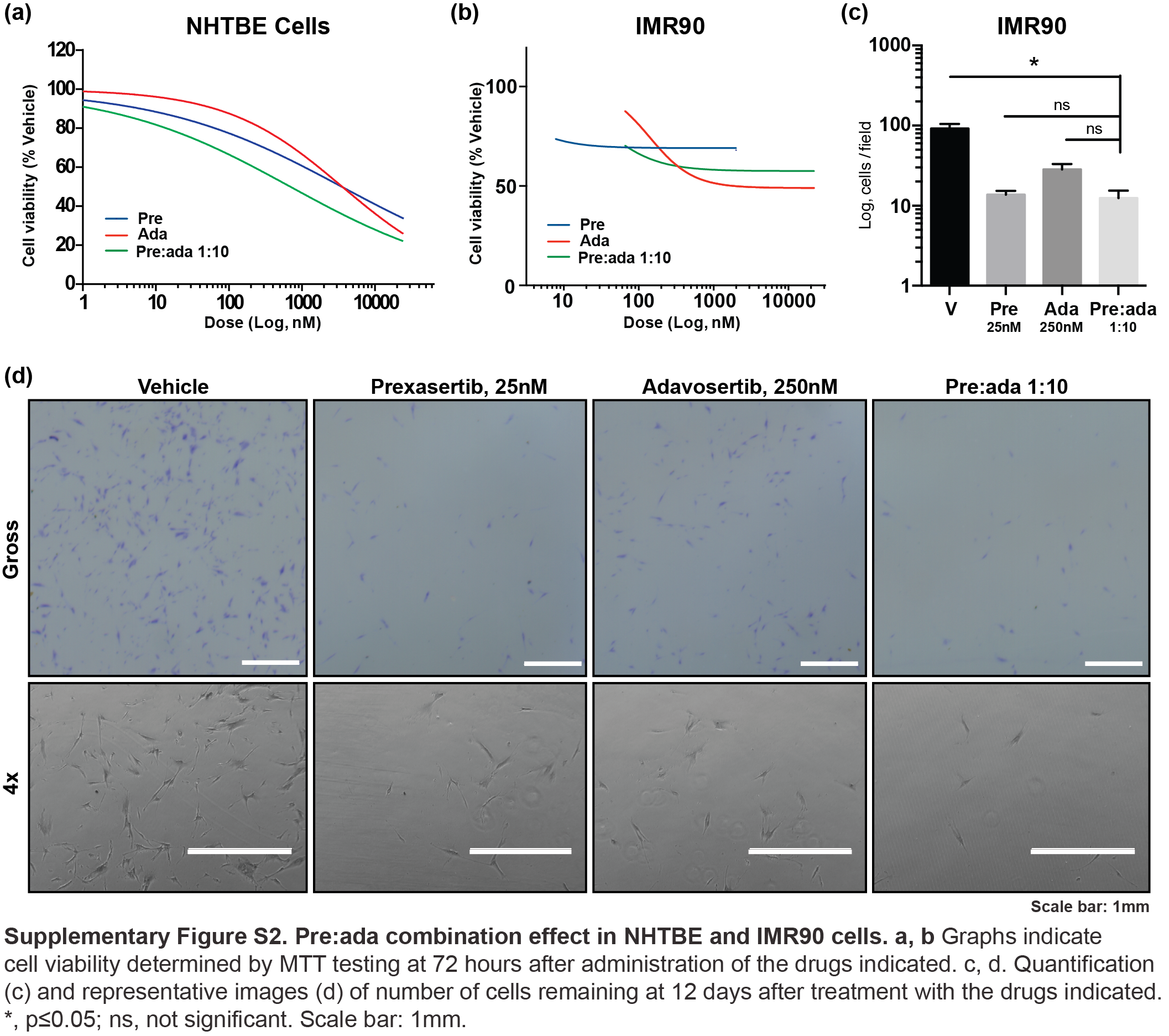

Supplement: Supplementary file 1 [file cancers-12-00306-s001.zip › cancers-672795-supplement-final/Supplementary Figure S2.png]

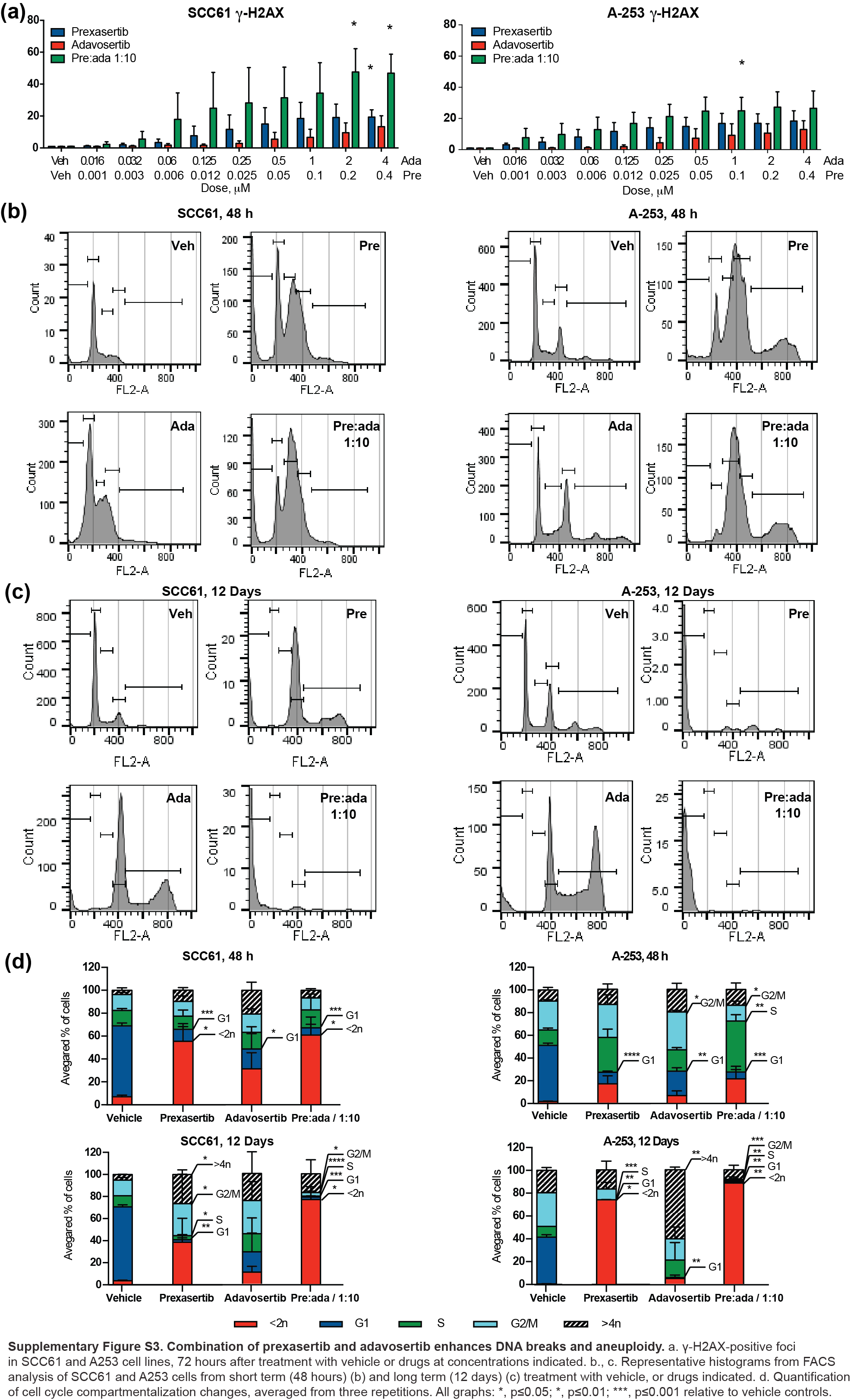

Supplement: Supplementary file 1 [file cancers-12-00306-s001.zip › cancers-672795-supplement-final/Supplementary Figure S3.png]
